# Epithelial V-Like Antigen Mediates Efficacy of Anti-Alpha_4_ Integrin Treatment in a Mouse Model of Multiple Sclerosis

**DOI:** 10.1371/journal.pone.0070954

**Published:** 2013-08-08

**Authors:** Erik Wright, Kusha Rahgozar, Nicholas Hallworth, Stefan Lanker, Michael D. Carrithers

**Affiliations:** 1 Department of Neurology, University of Wisconsin School of Medicine and Public Health, Madison, Wisconsin, United States of America; 2 Neurology Service, William Middleton VA, Madison, Wisconsin, United States of America; 3 Biogen-Idec, Cambridge, Massachusetts, United States of America; Hannover Medical School, Germany

## Abstract

Natalizumab inhibits the transmigration of activated T lymphocytes into the brain and is highly efficacious in multiple sclerosis (MS). However, from a pharmacogenomic perspective, its efficacy and safety in specific patients remain unclear. Here our goal was to analyze the effects of epithelial V-like antigen (EVA) on anti-alpha_4_ integrin (VLA4) efficacy in a mouse model of MS, experimental autoimmune encephalomyelitis (EAE). EVA has been previously characterized in human CD4 T lymphocytes, mouse thymic development, and choroid plexus epithelial cells. Further analysis here demonstrated expression in B lymphocytes and an increase in EVA^+^ lymphocytes following immunization. Following active induction of EAE using the MOG^35–55^ active immunization model, EVA deficient mice developed more severe EAE and white matter tissue injury as compared to wild type controls. This severe EAE phenotype did not respond to anti-VLA4 treatment. In both the control antibody and anti-VLA4 conditions, these mice demonstrated persistent CNS invasion of mature B lymphocyte (CD19^+^, CD21^+^, sIgG^+^), increased serum autoantibody levels, and extensive complement and IgG deposition within lesions containing CD5^+^IgG^+^ cells. Wild type mice treated with control antibody also demonstrated the presence of CD19^+^, CD21^+^, sIgG^+^ cells within the CNS during peak EAE disease severity and detectable serum autoantibody. In contrast, wild type mice treated with anti-VLA4 demonstrated reduced serum autoantibody levels as compared to wild type controls and EVA-knockout mice. As expected, anti-VLA4 treatment in wild type mice reduced the total numbers of all CNS mononuclear cells and markedly decreased CD4 T lymphocyte invasion. Treatment also reduced the frequency of CD19^+^, CD21^+^, sIgG^+^ cells in the CNS. These results suggest that anti-VLA4 treatment may reduce B lymphocyte associated autoimmunity in some individuals and that EVA expression is necessary for an optimal therapeutic response. We postulate that these findings could optimize the selection of treatment responders.

## Introduction

Immune therapy for patients with multiple sclerosis (MS) presents several challenges [1]. Although potential adverse events and route of administration clearly influence treatment decisions, relative efficacy and clinical response in specific individuals, the most important criteria for patients with active disease, can be difficult to assess [2].

Natalizumab is a humanized monoclonal antibody targeted to alpha_4_ integrin (VLA4) that blocks CD4 T lymphocyte transmigration into tissues including the brain parenchyma. This approach was developed through pre-clinical studies of VLA4 blockade in experimental autoimmune encephalomyelitis (EAE) [3] and is a highly efficacious treatment for relapsing forms of MS [4]. One drawback to natalizumab therapy is that it is associated with the development of progressive multifocal leukoencephalopathy (PML), particularly in those patients with a detectable humoral response to JC virus and prior immune suppression. PML is fatal in about 20% percent of patients in MS. The approximate risk of PML is less than 1 in 10,000 for patients with a negative anti-JCV titer and as high as 11 in 1000 for those with a positive titer and prior exposure to immunosuppressant medications [5].

The use of natalizumab does not appear to increase the risk of other infections associated with impaired CD4 T lymphocyte function. This observation suggests that a functional, but decreased, level of immune surveillance may remain intact during treatment in the majority of patients. During health, migration of CD4 T lymphocytes into the CNS is mediated in part by the choroid plexus epithelium at blood cerebrospinal fluid barrier and appears to be VLA4-independent [6], [7]. The mechanism of impaired immune surveillance in natalizumab-treated patients that develop PML remains unclear.

Recent studies suggested a role for epithelial V-like antigen (EVA) in the regulation of lymphocyte and neuroprotective barrier function during health and disease. EVA belongs to the immunoglobulin superfamily of proteins and is expressed in the thymus, choroid plexus epithelial cells, and mature CD4^+^ T lymphocytes [8], [9], [10]. Human peripheral blood CD4^+^EVA^+^ T lymphocytes express high levels of interleukin 22 and can interact directly with other EVA^+^ lymphocytes and choroid plexus epithelium through EVA homophilic interactions [10].

Here our goals were to assess the potential role of EVA in EAE pathogenesis and response to anti-VLA4 treatment. Consistent with its proposed regulatory roles, EVA deficient mice developed more severe clinical disease. In addition, we demonstrated that EVA expression is necessary for anti-VLA4 efficacy and unexpectedly identified a potential role for EVA in B lymphocyte function.

## Materials and Methods

### Ethics Statement

This study was carried out in strict accordance with the recommendations in the Guide for the Care and Use of Laboratory Animals of the National Institutes of Health. The protocols were approved by the Institutional Animal Care and Use Committee of the University of Wisconsin, Madison (Protocol numbers M024031 and M02544). All efforts were made to minimize suffering.

A detailed description of Materials and Methods is available in supplemental information (File S1).

## Results

### *In vivo* Expression of EVA in Naive and Immunized Mice

**Figure 1:**
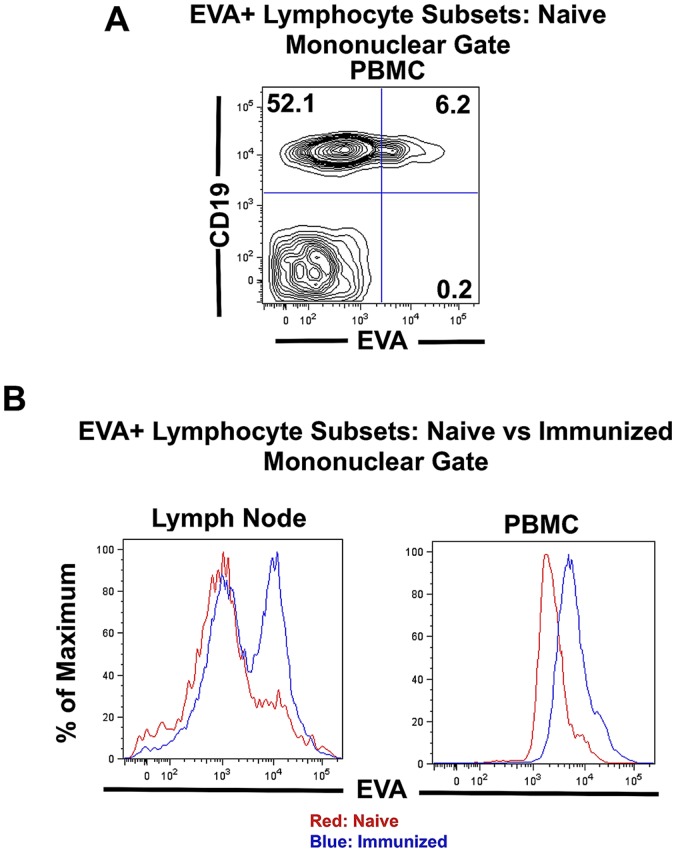
Characterization of EVA^+^ lymphocyte subsets *in vivo.* **A.**In naïve, unimmunized mice, approximately 3–9% of mononuclear cells express EVA (Table 1). A majority of these are CD19^+^ B lymphocytes. For flow cytometry analysis of lymphocyte subsets, gating was performed on the mononuclear subset as defined by FSC/SSC criteria. **B.** Following immunization with MOG^35–55^ using CFA and pertussis toxin as adjuvants, the percent of EVA^+^ lymphocytes prior to disease onset (day 12) increased markedly in lymph node and peripheral blood (Table 1).

Prior work from this laboratory demonstrated expression of EVA in mouse and human choroid plexus epithelial cells and in human CD4 T lymphocytes. Here we performed additional characterization of EVA expression in immune cells in naive and MOG^35–55^ immunized mice. In naive mice, approximately 3–9% of mononuclear cells (mononuclear gates as defined by FSC/SSC gating) in peripheral blood (peripheral blood mononuclear cells; PBMC) and lymph node are EVA^+^ (Table 1; n = 3–7 for each source). Surprisingly, we demonstrated that the majority of EVA^+^ mononuclear cells were CD19^+^ B lymphocytes and represented 65 to 94% of the EVA^+^ population (Table 1; Fig. 1A). For PBMC and lymph node, the percent of EVA^+^ B lymphocytes represented a relative increase as compared to total B cell percents in these tissues. To our knowledge, this observation represents the first demonstration of EVA expression in B cells.

**Table 1:** Analysis of EVA^+^ mononuclear cell subsets in C57BL6 mice.

Source	Condition	% EVA^+^ Cells (± SEM)	EVA^+^ Subsets	
			CD19	CD4
Peripheral Blood	Naive	4.6±0.9%	94.3±2.1%	0.4±0.3%
	Immunized	38.9±8.1%*	51.8±13.8%*	24.8±5.3%*
Lymph Node	Naive	3.0±0.7%	84.6±2.0%	5.6±1.1%
	Immunized	43.5±4.5%*	69.4±9.2%	10.1±3.2%

* *P*<0.05 as compared to the naive condition.

EVA^+^ CD4 T lymphocytes were detectable in lymph node but at a lower frequency; they were present at very low or undetectable levels in peripheral blood (Table 1). As previously shown by another laboratory, EVA^+^ T lymphocytes are present in the thymus (data not shown), consistent with its proposed role in T cell development [10].

We also examined EVA expression following immunization with MOG^35–55^ using CFA and pertussis toxin as adjuvants. In mononuclear cells from draining lymph node and peripheral blood, the percentage of EVA^+^ lymphocytes increased markedly (Fig. 1B; Table 1). As a percent of total mononuclear cells, EVA^+^ cells in peripheral blood increased from 4.6±0.9% to 38.9±8.1%, and in lymph node from 3.0±0.7% to 43.5±4.5% (*P*<0.05; n = 3 for the naive condition; n = 5–7 for the immunized condition). In peripheral blood, the majority of EVA^+^ mononuclear cells were CD19^+^ (51.8±13.8% of EVA+ cells and approximately 20% of total PBMC) as in the naive condition. However, immunization substantially increased the percent of CD4 cells in the EVA^+^ population. In the EVA^+^ PBMC population, EVA^+^CD4^+^ frequencies were 0.4±0.3% for the naive condition vs. 24.8±5.3% for the immunized condition. Although EVA^+^ lymphocytes were also identified in the spleen, the increase in EVA expression following immunization was less marked and not statistically significant (data not shown).

These results demonstrated that EVA is expressed on a subset of lymphocytes *in vivo* and suggested that it may represent an activation marker in B and T lymphocytes following immunization. The mechanistic significance of relative increases in EVA^+^ lymphocyte subset frequencies following immunization is unclear at this time.

### EVA-knockout Mice have Normal Immune System Development and Choroid Plexus Morphology

To study *in vivo* function of EVA, we characterized EVA-deficient mice (B6N.129S5-*Mpzl2^tm1Lex^*/Mmucd, Mutant Mouse Regional Resource Center at University of California, Davis) [11]. qPCR analysis of mRNA expression of EVA in the thymus confirmed absent expression in these mice. mRNA levels were 17.87±0.06 copies/GAPDH x10^−5^ in EVA^+/+^ and not detected in EVA^−/−^ as defined by C_t_>50 cycles (n = 3 for each condition).

Nevertheless, these mice have a normal lifespan, and flow cytometry analysis revealed normal cellular composition in the thymus, spleen, lymph node and peripheral blood (data not shown). Following immunization, there was no difference in VLA4 expression in lymphocyte subsets between wild type and EVA^−/−^ peripheral blood or lymph node.

Prior studies also demonstrated expression of EVA in mouse and human choroid plexus epithelial cells [9]. In mature wild type mice, EVA is expressed at the cell surface and intracellularly. In EVA knockout mice, the choroid plexus has a normal morphologic appearance in hematoxylin and eosin stained sections. None of the mice developed hydrocephalus or other spontaneous CNS disorder. These results suggested that EVA is not necessary for lymphoid or choroid plexus development.

### EVA Deficiency Results in Increased EAE Disease Severity

**Figure 2:**
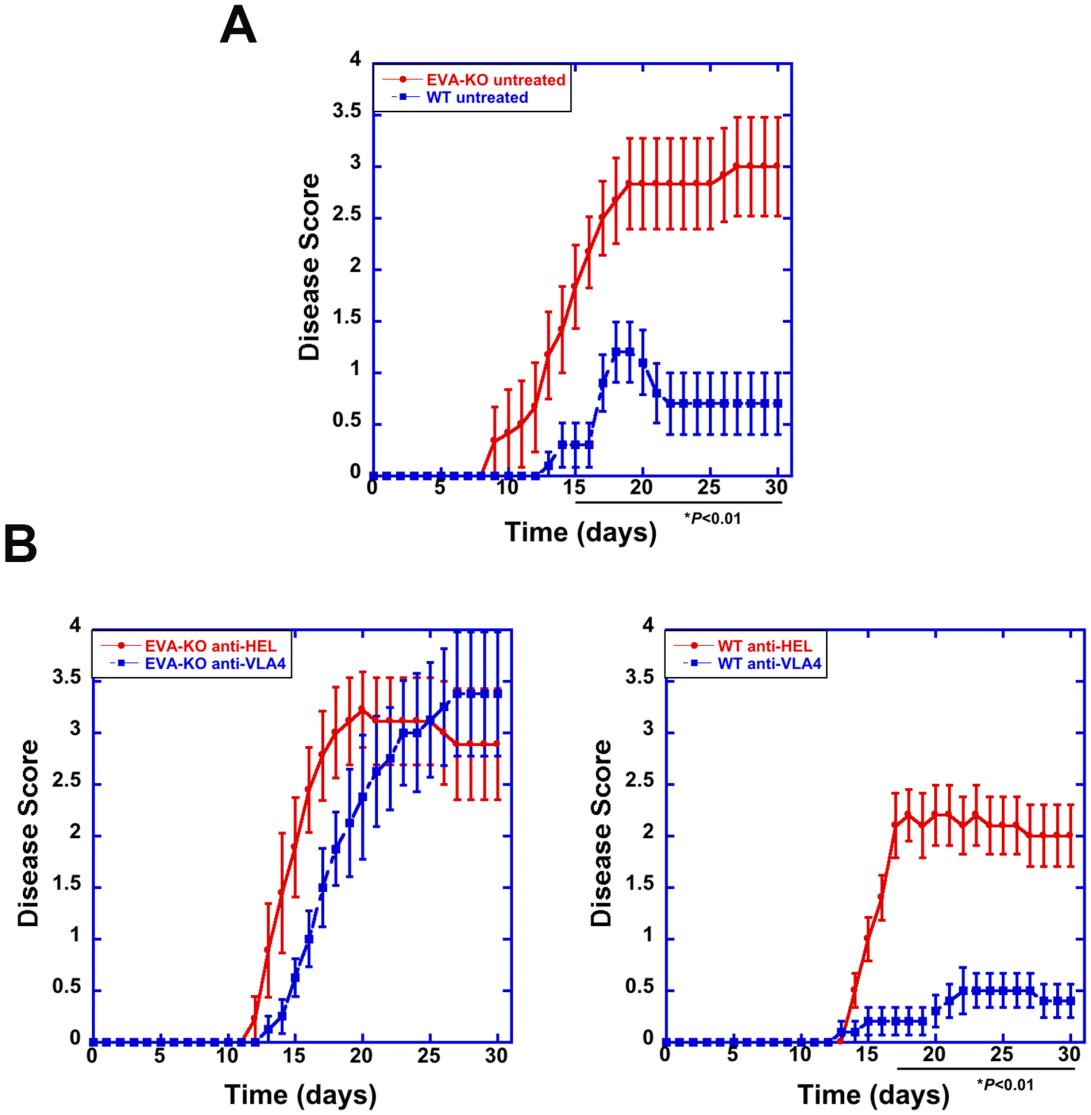
Increased EAE disease severity and resistance to anti-VLA4 treatment in EVA-deficient mice. EAE was induced by active immunization at 2 sites with MOG peptide^35–55^ emusified in CFA (0.1 mL of emulsion/site; 1 mg/ml MOG^35–55^ and 2.5 mg/ml H37Ra killed mycobacterium). Pertussis toxin (100 ng ip) was administered on days 0 and 1. Age and sex-matched control littermates were used for the wild type group. Approximately 50% of the mice used for each condition were male. Animals were observed on a daily basis for signs of clinical EAE. The animals were graded as follows: 1, limp tail; 2, partial hind limb paralysis; 3. complete hind limb paralysis; 4, hind and front limb paralysis; 5, moribund. Animals were cared for in accordance with University of Wisconsin guidelines. **A.** As compared to sex and age-matched littermate wild type controls, EVA-deficient mice developed more severe EAE as determined by analysis of daily, cumulative and peak disease scores. Cumulative disease score was 48.4±8.2 in knockout mice (n = 12) and 12.5±4.3 in wild type (n = 10; *P*<0.01); peak disease score was 3.08±0.47 in knockouts and 1.30±0.30 in wild type (*P*<0.01). Differences in daily disease scores were statistically significant from day 15 through day 30, the final day of observation. **B.** In separate experiments, mice were treated with 4 mg/kg of either IgG_2_ isotype control anti-HEL (hen egg lysozyme) or anti-mouse VLA4 (PS/2 monoclonal antibody) intraperitoneally every 4 days for 28 days starting at day 0. As expected, wild type litter-mate controls treated with anti-VLA4 had significantly reduced disease severity as compared to anti-HEL-treated mice. Cumulative disease scores were 31.8±4.1 in the control anti-HEL treatment group versus 6.1±2.3 in the anti-VLA4 group (*P*<0.01, n = 10 for both groups) and, peak disease scores were 2.50±0.22 and 0.60±0.22, respectively (*P*<0.01). Differences in daily disease scores were statistically significant from day 17 through day 30. EVA-deficient mice were resistant to anti-VLA4 treatment and developed severe disease independent of treatment. Cumulative score was 49.1±7.8 in the control anti-HEL group and 41.1±6.4 in the anti-VLA4 group (*NS*; n = 9 for anti-HEL and n = 8 for anti-VLA4). Peak disease score was 3.22±0.36 in the anti-HEL control group and 3.38±0.60 in the anti-VLA4 group (*NS*).

We used the anti-MOG^35–55^ active immunization model to assess EAE disease course in EVA knockout mice. In immunized, untreated mice, EVA knockout mice demonstrated increased cumulative and peak disease severity as compared to wild type mice (Fig. 2A). Cumulative disease score was 48.4±8.2 in knockout mice (n = 12) and 12.5±4.3 in wild type (n = 10; *P*<0.01); peak disease score was 3.08±0.47 in knockouts and 1.30±0.30 in wild type (*P*<0.01). Differences in daily disease scores were statistically significant from day 15 through day 30, the final day of observation. These results suggested that EVA mediates a protective role in EAE pathogenesis.

### EVA Knockout Mice are Resistant to Anti-VLA4 Treatment

To assess the role of EVA in therapeutic response, we also examined anti-VLA4 treatment in EVA-deficient mice. In these experiments, mice received anti-VLA antibody or control anti-HEL antibody beginning on the day of immunization and every 4 days subsequently (4 mg/kg ip; a total of 8 injections). As expected, wild type mice treated with anti-VLA4 demonstrated a reduction in cumulative and peak disease score as compared to control, anti-HEL antibody, treated mice (Fig. 2B). Cumulative disease scores were 31.8±4.1 in the control anti-HEL treatment group versus 6.1±2.3 in the anti-VLA4 group (*P*<0.01, n = 10 for both groups), and peak disease scores were 2.50±0.22 and 0.60±0.22, respectively (*P*<0.01). Differences in daily disease scores were statistically significant from day 17 through day 30.

In contrast, EVA-deficient mice did not demonstrate a therapeutic response to anti-VLA4 treatment. There was a slight trend to delayed disease onset that did not result in a statistically significant change in cumulative or peak disease score. Cumulative score was 49.1±7.8 in the control anti-HEL group and 41.1±6.4 in the anti-VLA4 group (*NS*; n = 9 for anti-HEL and n = 8 for anti-VLA4). Peak disease score was 3.22±0.36 in the anti-HEL control group and 3.38±0.60 in the anti-VLA4 group (*NS*). These results suggested that EVA expression is necessary to achieve a full therapeutic response to anti-VLA treatment.

### Flow Cytometry Analysis of Spinal Cord Infiltrates

**Figure 3:**
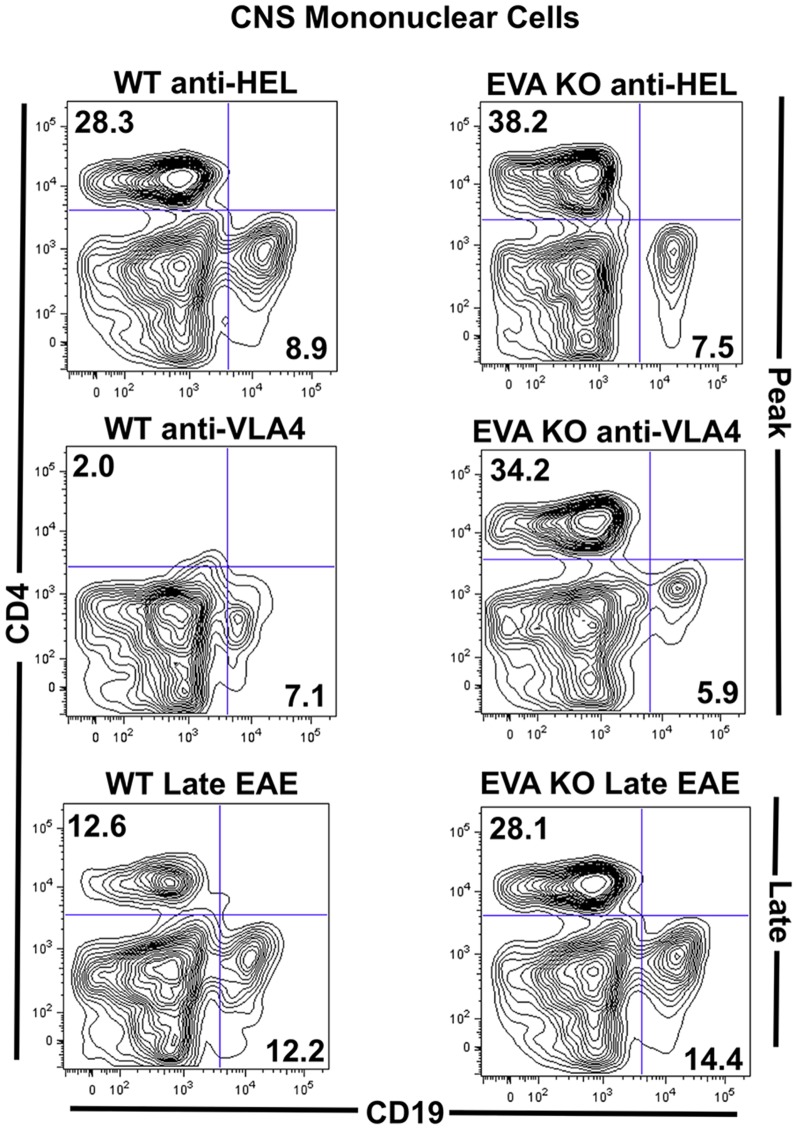
EVA-deficient mice demonstrate VLA4-independent CNS invasion during EAE. Flow cytometry analysis of mononuclear subsets from spinal cords of mice at onset of peak EAE (day 19) was performed. **A–B.** As predicted, total mononuclear cell counts (left y-axis) and percent CD4 invasion (right Y-axis) were reduced in wild type, litter-mate control mice treated with anti-VLA4. EVA-deficient mice demonstrated similar immune cell recruitment independent of treatment with control anti-HEL antibody or anti-VLA4. **C.** Percent differences in CD4, CD8, CD11b and CD19 populations were less marked between groups. There was a modest decrease in CD11b^+^ cells in spinal cords of EVA^−/−^ control treated mice as compared to the wild type control treatment group. Representative FACS plots are shown. Analysis of pooled data from multiple experiments is shown in Table 2.

To assess for potential differences in the composition of spinal cord inflammatory infiltrates between groups, we performed flow cytometry analysis. As expected, anti-VLA4 treated wild type mice had a significantly reduced number of total mononuclear cells and percent CD4^+^ cells in spinal cord single cell preparations (Fig. 3; Table 2). In addition, cytometric analysis demonstrated similar invasion of mononuclear cells into EVA-deficient mice treated with anti-VLA4 as compared to control treated EVA-deficient and wild type mice. Analysis of CD4, CD8, CD19, and CD11b subsets suggested similar composition of the inflammatory infiltrates in all groups that developed more severe EAE. The only trend noted was a modest decrease in CD11b^+^ cells in spinal cords of EVA^−/−^ control treated mice as compared to the wild type control treatment group. All CNS invasive T and B cells expressed VLA4, and there was no difference in expression between wild type and EVA-deficient mice. These results suggested that VLA4-independent recruitment of immune cells can occur during EAE in EVA-deficient mice. However, these findings did not suggest a mechanism to explain increased disease severity due to EVA deficiency.

**Table 2:** Analysis of CNS mononuclear cell subsets at onset of peak EAE (day 19).

Genotype	Treatment	%CD4 (±SEM)	%CD8 (±SEM)	%CD11b (± SEM)	%CD19 (± SEM)	sIgG Subsets	
						CD19^hi^ sIgG^hi^	CD19^int^sIgG^int^
EVA^−/−^	anti-HEL	29.2±2.6	7.5±1.0	28.5±3.9*	5.8±0.9	7.0±1.0	10.7±1.7
	anti-VLA4	26.0±2.9	5.5±1.3	27.4±5.8	5.7±0.2	5.7±0.3	11.8±1.7
EVA^+/+^	anti-HEL	31.5±1.9*	5.9±0.8	42.6±1.8*	4.9±1.1	8.4±0.3	2.0±0.4*
	anti-VLA4	10.4±2.2*	6.5±1.0	33.7±1.3	3.2±0.4	2.5±0.2*	2.0±0.6*

*P<0.01, *P<0.05, *P<0.01 (EVA^+/+^ anti-HEL vs anti-VLA4) (as compared to EVA^−/−^).

Total mononuclear cells counts per spinal cord were 73,143±8951 (EVA^−/−^ anti-HEL); 62,102±4008 (EVA^−/−^ anti-VLA4); 63,732±7504* (EVA^+/+^ anti-HEL); 17,540±2717* (EVA^+/+^ anti-VLA4).

* *P*<0.01 (EVA^+/+^ anti-HEL vs EVA^+/+^ anti-VLA4).

Data were generated from using multiple animals (3–7) from multiple independent experiments (3–4).

### Distinct Appearance of EAE Spinal Cord Lesions in Treated and Untreated EVA-Knockdown Mice

**Figure 4:**
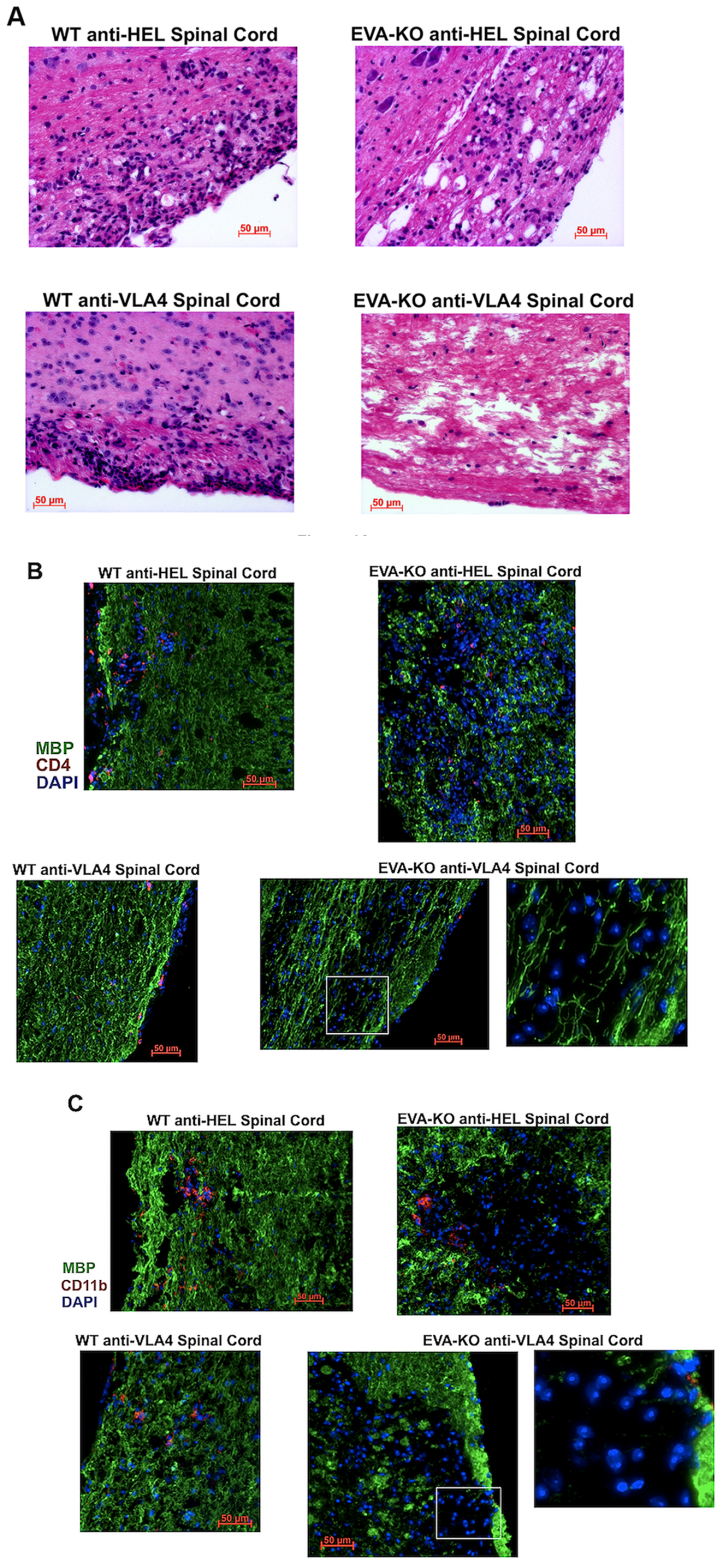
Distinct appearance of parenchymal spinal cord lesions in EVA-deficient mice during EAE. **A.** Hematoxylin and eosin stained sections of spinal cord lesions at onset of peak disease (day 19) demonstrated meningeal immune cell infiltration in all genotype and treatment conditions (longitudinal sections in lateral white matter). Scale bars, 50 µm**. B, C.** Immunofluorescence staining of longitudinal spinal cord sections in EVA-deficient conditions revealed parenchymal lesions devoid of myelin basic protein (MBP; green) associated with small numbers of CD4 and CD11b cells (red) and larger numbers of cells with blastic-appearing nuclei (DAPI nuclear stain; blue) that contained prominent nucleoli. Wild type lesions in control antibody treated mice demonstrated parenchymal invasion adjacent to meningeal infiltrates with CD4 and CD11b cells distributed throughout. A small number of meningeal inflammatory infiltrates were seen in anti-VLA4 treated mice. Scale bars, 50 µm.

To further examine potential mechanisms of increased disease severity and resistance to anti-VLA4 treatment, we performed histological analysis of spinal cord inflammatory infiltrates. Consistent with the flow cytometry findings (Fig. 3), EVA^−/−^ mice with EAE did not demonstrate an increase in CD4^+^ and CD11b^+^ cell numbers within spinal cord inflammatory infiltrates as compared to wild type mice treated with control anti-HEL antibody (Fig. 4).

However, consistent with increased disease severity (Fig. 2), histological analysis did reveal a distinct appearance of parenchymal lesions in EVA^−/−^ mice from both treatment conditions as compared to wild type treatment groups. Wild type parenchymal lesions were primarily observed adjacent to the meninges (Figs. 4A). Although anti-VLA4 treatment reduces total CNS mononuclear cell counts substantially as demonstrated by flow cytometry analysis (Fig. 3; Table 2), rare inflammatory infiltrates in the meninges were observed. In anti-HEL treated wild type mice, there was extension of infiltrates from the meninges into the spinal cord parenchyma. These lesions demonstrated numerous CD4^+^ and CD11b^+^ cells distributed throughout the inflammatory infiltrates (Fig. 4B).

In EVA^−/−^ spinal cord, some lesions were adjacent to the meninges as seen in wild type (Fig. 4A). However, parenchymal lesions in the absence of marked local meningeal infiltrates also were observed (Fig. 4BC). In these lesions, CD4^+^ cells were present, but more sparsely distributed as compared to wild type lesions, and CD11b^+^ cells were primarily localized to the leading edge of regions of demyelination. In addition, there were many mononuclear cells with prominent nucleoli within these lesions in EVA^−/−^ mice. These cells did not stain positive for either T cell (CD4, CD8) or myeloid markers (CD11b, F4/80). The distinct appearance of the lesions in EVA^−/−^ mice from both treatment conditions also was associated with extensive loss of staining for myelin basic protein (MBP). The relatively low number of lesional CD4^+^ cells, CD11b^+^ cells limited to the leading edge of demyelination, and the presence of cells with a blastic appearance that lacked expression of T cell and myeloid markers suggested that the severe EAE disease phenotype observed in anti-HEL and anti-VLA4 treated EVA^−/−^ mice may be due to T cell-independent mechanisms.

### Inflammatory Changes in the Choroid Plexus

Since EVA is not only expressed in lymphocytes but also by choroid plexus epithelial cells, we examined choroid plexus morphology during EAE in all treatment groups. All immunized mice, independent of disease score, demonstrated choroid plexus inflammatory infiltrates and evidence of impaired barrier function (data not shown). All groups showed reduced retention of intravascular fluorescently-labeled dextran within the choroid plexus as compared to healthy controls. This finding suggested either increased leakage of tracer or reduced perfusion. Although we cannot exclude a role in disease initiation, these results suggested that increased disease severity and treatment resistance associated with EVA deficiency is not primarily due to impaired choroid plexus function.

### Enhanced Local and Systemic Humoral Responses during EAE in EVA-knockout Mice

**Figure 5:**
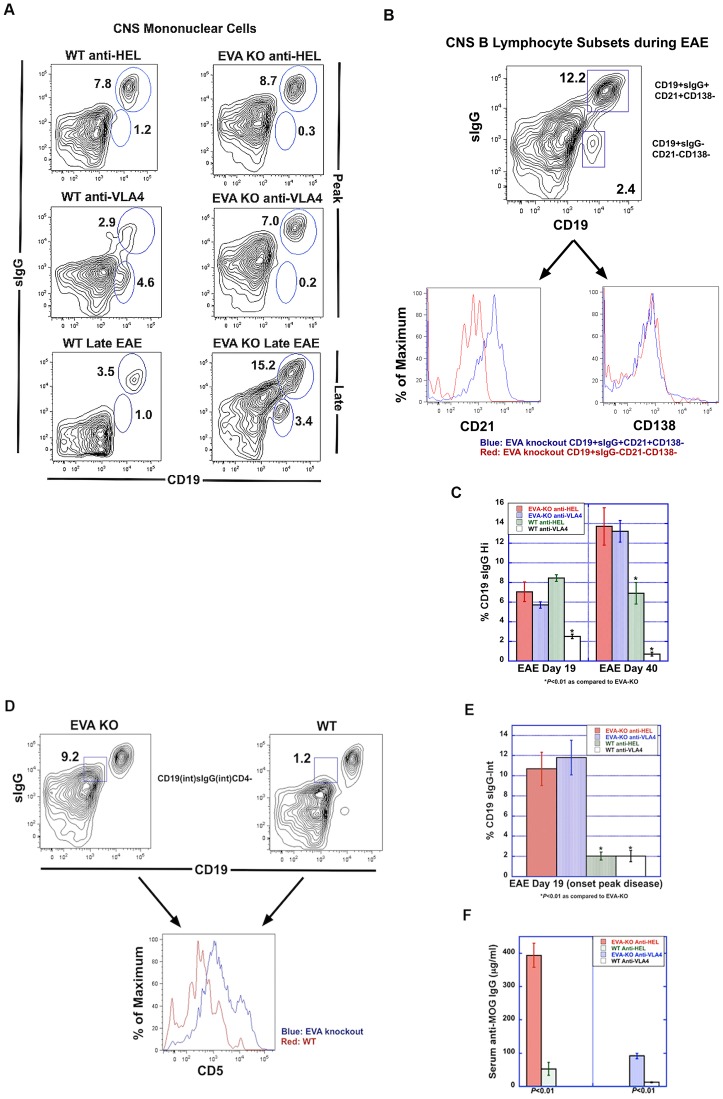
Analysis of humoral responses during EAE. **A.** Flow cytometry analysis was performed to examine mature B cells within spinal cord infiltrates. Cells were stained for expression of CD19 and surface IgG (sIgG) and analyzed by flow cytometry. At the onset of peak disease (day 19), anti-VLA4 treatment reduced CNS invasion in wild type, but not EVA-deficient mice. At a late stage of EAE (day 40), EVA-deficient mice demonstrated persistence of high levels of mature CD19^+^sIgG^+^ (surface IgG-positive) B lymphocytes. Representative FACS plots are shown. Analysis of pooled data from multiple experiments are shown in Tables 2–3 and the bar graphs. **B–C.** B cell subset analysis in EAE spinal cord revealed that the CD19^+^sIgG^+^ population shown in **A** expresses high levels of the maturation marker, CD21, but not the plasma cell marker, CD138. Quantitative analysis revealed persistence of mature B cells (Table 3 and bar graph) in EVA-deficient mice as compared to wild type litter- mates (Table 3 and bar graph). At onset of peak disease, anti-VLA4 treatment reduced invasion of this population in wild type but not EVA-deficient mice. **D–E.** We also identified a population of CD19^int^sIgG^int^ cells from EVA-deficient mice that was increased in spinal cord infiltrates as compared to both control treatment conditions at onset of peak disease (Table 2 and bar graph). In EVA-deficient mice, most of these cells expressed intermediate and high levels of CD5. **F.** Serum autoantibody levels (anti-MOG IgG) were measured at onset of peak disease by ELISA. EVA-deficient mice demonstrated increased autoantibody levels as compared to wild type mice from the same treatment condition. Anti-VLA4 treatment prevented the development of autoantibody in wild type mice. Although treatment appeared to reduce levels in EVA-deficient mice, autoantibody serum concentration remained higher than that seen in wild type control treated mice. At onset of peak EAE, serum IgG anti-MOG levels were 393.4±36.1 µg/ml in the knockout anti-HEL treated group, 91.6±8.7 µg/ml in knockout anti-VLA4, 52.3±19.5 µg/ml in wild type anti-HEL and 12.2±1.7 µg/ml wild type anti-VLA4.

Based on the expression of EVA in large numbers of B lymphocytes and the presence of blastic-appearing cells in CNS lesions from EVA^−/−^ mice that lacked T cell and myeloid markers, we hypothesized that humoral responses mediated, in part, increased disease severity in the knockout condition. Flow cytometry analysis of mononuclear cell spinal cord infiltrates at onset of peak disease (Day 19) and late EAE (Day 40) demonstrated the presence of mature B lymphocytes as defined as surface IgG-positive CD19^+^ cells (CD19^+^sIgG^+^) cells in all treatment conditions (Fig. 5A). At onset of peak disease, anti-VLA4 treatment reduced CNS infiltration of CD19^+^sIgG^+^ cells in wild type but not EVA-deficient mice. These cells persisted in the CNS of EVA-deficient mice through day 40 but did so to a lesser extent in control treated wild type mice.

Phenotypic analysis of B cell subtypes within the CNS during EAE revealed the absence of CD138^+^ plasma cells in any of the treatment conditions at onset of peak disease and in the late EAE disease stage (Fig. 5BC). Invasive CD19^+^sIgG^+^ cells also expressed the B cell maturation factor CD21, which was not observed in the CD19^+^sIgG^lo^ population. The latter population was predominant in VLA4-treated wild type mice.

Additional flow cytometry analysis of spinal cord mononuclear cells demonstrated the presence of a CD19^int^sIgG^int^CD4^−^ population at onset of peak disease and in late EAE in EVA-deficient mice treated with either control antibody or anti-VLA4 (Fig. 5DE; Table 3). This population was either not detectable or present at very low levels in wild type mice from both treatment conditions. In EVA-deficient mice, these cells expressed intermediate and high levels of CD5, a marker of B-1a lymphocytes; CD5 expression in this population was lower in wild type mice.

**Table 3:** Analysis of CNS mature B cells during late EAE (day 40).

Genotype	Treatment	%CD19^hi^sIgG^hi^ (±SEM)
EVA^−/−^	anti-HEL	13.7±1.9
	anti-VLA4	13.2±1.1
EVA^+/+^	anti-HEL	6.9±1.1*
	anti-VLA4	0.7±0.2*

* *P*<0.01 (as compared to EVA^−/−^).

Data were generated from using multiple animals (3–4) from multiple experiments (3).

These findings demonstrated that EVA-deficient mice have increased numbers of CD5^+^sIgG^int^CD19^int^ and persistent presence of mature CD19^+^sIgG^+^CD21^+^CD138^−^ cells within the CNS throughout the course of EAE. These results suggested a potential humorally-mediated mechanism to explain the increased EAE severity and lack of treatment response in EVA-deficient mice.

The continued presence of mature CD19^+^sIgG^+^CD21^+^CD138^−^ cells within the CNS of EVA-deficient mice suggests a potential mechanism that may mediate a more chronic, progressive disease process.

### Increased Serum Autoantibody in EVA-deficient Mice during EAE

To assess the systemic humoral response during EAE, we performed serum analysis for the presence of anti-MOG IgG (Fig. 5F). At onset of peak EAE, serum IgG anti-MOG levels were 393.4±36.1 µg/ml in the knockout anti-HEL treated group, 91.6±8.7 µg/ml in knockout anti-VLA4, 52.3±19.5 µg/ml in wild type anti-HEL and 12.2±1.7 µg/ml wild type anti-VLA4. These results suggested that anti-VLA4 treatment can reduce autoantibody levels during EAE and that EVA deficiency may enhance autoantibody production.

### Inflammatory Spinal Cord Lesions from EVA-deficient Mice with EAE Demonstrate Increased Numbers of IgG^+^ Cells, Antibody and Complement Deposition, and B cell Phenotypic Heterogeneity

As described above (Fig. 4), immunofluorescent staining of spinal cord lesions demonstrated distinct differences between anti-HEL control treated wild type mice and EVA^−/−^ mice treated with either anti-HEL or anti-VLA4. EVA-deficient mice showed enlarged lesions with many blastic-appearing cells that were CD4 and CD11b negative. We further analyzed lesions by immunofluorescence to assess B lymphocyte subtypes.

**Figure 6:**
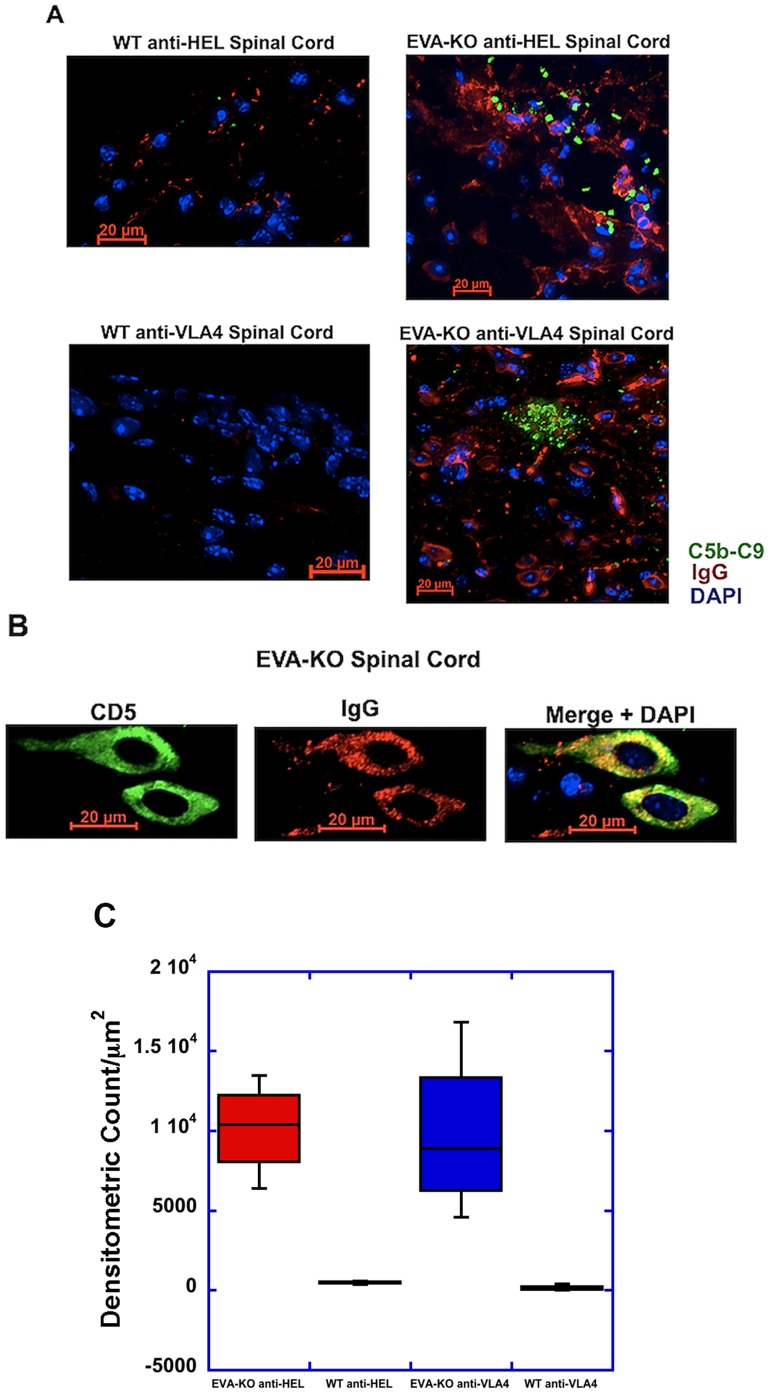
Increased parenchymal IgG and complement deposition in lesions from EVA-deficient mice. **A.** At onset of peak disease (day 19), EVA-deficient mice from both treatment conditions demonstrated the presence of numerous IgG^+^ cells with a similar nuclear appearance to those cells shown in Figure 4. Similar levels of antibody (red) and complement deposition (green) were not seen in control mice. **B.** A higher power view of the cells shown in (A) shows the presence of high levels of cytoplasmic IgG (red) in CD5^+^ (green) cells. Scale bars, 20 µm**. C.** Quantitative analysis of complement deposition showed marked differences between the EVA-deficiency treatment groups and the wild type. 3–4 spinal cords were analyzed per condition. The densitometric counts per µm^2^ were 10,170±1473 relative fluorescent units (RFU’s) for the anti-HEL treated EVA^−/−^ group (n = 3), 485±71 RFU’s for the anti-HEL wild type group (n = 4; P<0.05 compared to EVA^−/−^), 9804±2586 RFU’s for the anti-VLA4 treated EVA^−/−^ (n = 4), and 139±94 for the anti-VLA wild type group (n = 4; P<0.05 compared to EVA^−/−^). In the spinal cords analyzed, there were 1075 discrete regions of positive staining for complement identified in the anti-HEL knockouts, 41 in the anti-HEL wild type, 334 in the anti-VLA4 knockout, and 18 in the anti-VLA4 wild type.

In lesions from knockout mice of both treatment conditions, many of the blastic-appearing cells seen in figures 4B and 4C stained positive for surface and cytoplasmic IgG, and there were extensive amounts of non-cell associated IgG deposition within the tissue (Fig. 6A). In addition, there was marked complement deposition within these parenchymal lesions that was not observed in the wild type mice from either treatment condition. Many of the IgG^+^ cells were CD5^+^ (Fig. 6B). Quantitative analysis of these lesions confirmed extensive complement deposition in the EVA-deficiency conditions but not in the wild type (Fig. 6C; result shown in figure legend). These findings suggested that the increased tissue injury and impaired therapeutic response observed in EVA-deficient mice is associated with CNS deposition of IgG, complement-mediated mechanisms, and CNS invasion of CD5^+^IgG^+^ cells in addition to other B cell populations.

## Discussion

We demonstrate that EVA regulates EAE disease phenotype and clinical response to anti-VLA4 therapy. Our findings show that EVA knockdown increases disease severity, prevents a clinical response to an anti-VLA4 monoclonal antibody, and is associated with markers of B cell-dependent injury. This work suggests a role for anti-VLA4 treatment in the prevention of mature B cell CNS invasion and autoantibody-mediated injury in some individuals with CNS inflammatory demyelinating disease.

In addition, our studies revealed expression of EVA in B lymphocytes and an increase in blood and draining lymph node of EVA^+^ lymphocytes following immunization. Although prior work had shown that EVA is expressed in T lymphocytes and epithelial cells, to our knowledge, this study is the first to demonstrate a potential role for EVA in B cell function. These observations reveal a potentially important role for EVA in the regulation of systemic and tissue-specific B cell responses.

Our results in EAE studies suggest that EVA limits the magnitude of systemic immune responses and subsequent tissue injury. The effect of EVA deficiency appears to be primarily mediated by an enhanced and more heterogeneous B cell response associated with persistence of mature B cells within the CNS, increased autoantibody production and CNS invasion of CD5^+^IgG^+^ cells. The presence of mature CD19^+^sIgG^+^CD21^+^CD138^−^ cells at onset of peak disease did not appear to be sufficient for the development of more severe EAE (Fig. 5); however, anti-VLA4 treatment in wild type mice that were protected from disease prevented CNS invasion of this cell subset. These results support a role for mature B cells in EAE pathogenesis and suggest a mechanism of action of anti-VLA4 treatment in addition to inhibition of T cell trafficking.

Persistence of mature B cells and plasma cells within the CNS may provide a source of oligoclonal production of antibodies and mediate conversion of relapsing MS to secondary progressive disease [12]. Although mature B cells may contribute to the disease pathogenesis in our EVA deficiency model as a source of autoantibody or as an antigen presenting cell, they did not appear to be present in large numbers at the sites of CNS lesions. Rather, the lesions contained large numbers of CD5^+^ cells that expressed intermediate levels of cell surface IgG (sIgG) and large amounts of cytoplasmic IgG (Figs. 5, 6). These B cells likely represent a population of innate cells, designated B-1a [13], that usually express IgM natural antibodies but can undergo maturation to produce IgG [14]. They have been hypothesized to mediate autoimmune pathogenesis in a variety of conditions [15]. To our knowledge, high VLA4 expression in this B cell subset, including CD5^+^ B cell neoplasms [16], has not been reported, and we postulate that they enter the CNS by a VLA-independent mechanism.

Our findings suggest a complex interplay between classical mature B lymphocytes and B1-a cells in the pathogenesis of EAE in EVA-deficient mice. These mechanisms are consistent with prior studies that have demonstrated CNS persistence of mature B cells [17] and increased numbers of peripheral [18] and CSF [19] CD5^+^ B cells in MS patients with secondary progressive and active relapsing disease. However, their specific mechanistic relationship to the development and relative disease severity in MS is unclear and requires further characterization.

Additional mechanisms that could contribute to the disease phenotype during EVA deficiency include T cell-dependent and CSF-barrier pathways. In those knockdown mice treated with anti-VLA4 antibody, there was VLA4-independent homing of CD4 T lymphocytes to the CNS. Since EVA can function as a cell surface adhesion molecule, one possibility is that it could limit homing to target organs. However, the degree of mononuclear cell infiltration during EAE did not differ between control treated wild type mice and the two knockout conditions. In addition, we have observed EVA^+^ lymphocytes within the CNS of wild type mice with clinical disease. This result would not be expected if EVA inhibited cell migration.

A second possibility is that EVA deficiency leads to a defective blood-CSF barrier, which may affect disease initiation, even if it does not enhance subsequent immune cell recruitment. However, both wild type and knockdown mice demonstrated similar morphological findings in choroid plexus during disease, even in wild types treated with anti-VLA4. It is possible that EVA deficiency permits a greater degree of VLA4-independent homing to the inflamed spinal cord of knockdown mice treated with anti-VLA4. However, this mechanism cannot explain the enhanced peripheral and central humoral response or the difference in disease severity between untreated or control-treated wild type and knockdown mice.

The potential role of EVA in B cell maturation suggests that it may be relevant to the pathogenesis of PML in natalizumab-treated patients. B cells can be infected with JC virus and serve as a latent reservoir [20]. In addition, a recent study of MS patients demonstrated that prolonged treatment with natalizumab leads to a higher percentage of memory and marginal zone-like B lymphocytes [21]. In susceptible individuals, a defect in adhesion molecule interactions, potentially including EVA-dependent mechanisms, may lead to enhanced B cell mobilization from the bone marrow, a known reservoir for JC virus [22].

It remains unclear whether or not EVA deficiency best models an aggressive form of MS associated with an enhanced humoral response or a related disease process such as neuromyelitis optica (NMO). This issue has clinical relevance for treatment decisions in patients with central demyelinating disease because the treatment efficacy differs between MS and NMO. Natalizumab does not appear to have efficacy in NMO [23], and type 1 interferons may exacerbate the disease [24].

Nevertheless, in the model of EAE used here, our data demonstrate that anti-VLA4 treatment in wild type mice prevents the development of an autoantibody response, CNS invasion of mature B cells, and subsequent tissue injury. This mechanism may represent an underappreciated therapeutic effect of natalizumab. In addition, other investigators have identified MS patients that are not optimal responders to natalizumab [2], and there may be subsets of patients with EVA deficient signaling that prevents a therapeutic response or increases the risk of the development of PML.

## Supporting Information

File S1 **Supplementary Information**
(DOCX)
