# Supplementary material for: Epithelial V-Like Antigen Mediates Efficacy of Anti-Alpha4 Integrin Treatment in a Mouse Model of Multiple Sclerosis
Source: PLoS One. 2013 Aug 8;8(8):e70954. doi: 10.1371/journal.pone.0070954 (PMC3738635; doi:10.1371/journal.pone.0070954)
Supplement: File S1 — Supplementary Information (DOCX) [file pone.0070954.s001.docx]

Supplementary Information

Materials and Methods

*Mice.* Experimental mouse strains were bred at our on-campus breeding facility (Biotron), where our transgenic colony is maintained. Experimental mice were transferred to an approved University of Wisconsin-Madison animal facility for the performance and monitoring of all *in vivo* experiments. EVA-knockout mice (B6N.129S5-*Mpzl2^tm1Lex^*/Mmucd) backcrossed for greater than 10 generations on the C57BL5 background were obtained from the Mutant Mouse Regional Resource Center at University of California, Davis [1].

Age (12-16 weeks of age) and sex (approximately similar numbers of males and females in each group) matched EVA knockout and wild type littermates were used for immune phenotype and EAE experiments. 8-12 mice were used for each EAE condition, and 3-7 mice for each histological and flow cytometry condition (same age and sex ratios as for EAE experiments).

*EAE induction*. A commercially available kit (Hooke Laboratories) was used to induce EAE. Mice were immunized with MOG^35-55^ (myelin oligodendrocyte glycoprotein) peptide emulsified in complete Freund’s adjuvant (CFA) by injecting them subcutaneously at two sites on the back (0.1 mL of emulsion/site; 1 mg/ml MOG^35-55^ and 2.5 mg/ml H37Ra killed mycobacterium). On the same and subsequent days, an intra-peritoneal injection of pertussis toxin in PBS, at 100 ng/mouse/dose (0.1 mL), was performed. Mice were examined daily while they were sick and, based on how sick they are, were given additional soft food (Dough Diet) and a hydration source (Napa Nectar) in the bottom of their cage.

Mice were followed for up to 40 days. Using this protocol in our colony, onset of disease usually occurs at approximately day 14 and onset of peak disease occurs 5 days following disease onset (onset of peak disease, average is day 19). Animals were observed on a daily basis for signs of clinical EAE. The animals were graded by a blinded examiner as follows: 1, limp tail; 2, partial hind limb paralysis; 3. complete hind limb paralysis; 4, hind and front limb paralysis; 5, moribund. If the mice developed a score of 4 (quadriparesis), they were euthanized.

*Treatment.* For some EAE experiments, mice were treated with 4 mg/kg of either IgG_2_ isotype control anti-HEL (hen egg lysozyme) or anti-mouse VLA4 (PS/2 monoclonal antibody) intraperitoneally every 4 days for 28 days starting at day 0. A subset of mice used for flow cytometry analysis were treated through day 40.

*Histology.* Perfusions, tissue preparation for frozen sections, sectioning, and staining for immunofluorescence were performed as described in our prior work [2,3]. For some experiments, dextran-FITC (40kDa; Sigma) was administered iv (0.1 ml of a 100 mg/ml solution in PBS) 2 min prior to perfusions.

For perfusions, mice were deeply anesthetized with ketamine/xylazine. Intracardiac perfusions were performed with ice cold PBS followed by 4% paraformaldehyde. Tissue was fixed overnight in 4% paraformaldehyde. For frozen sections for immunofluorescence, samples were cryopreserved in sucrose solutions and embedded in OCT.

For hematoxylin and eosin stains, tissue was dehydrated through graded ethanol, cleared in xylene, and embedded in paraffin. Processing and staining for hematoxylin and eosin staining were performed at the University of Wisconsin Department of Pathology core facility.

For immunofluorescence staining, frozen sections (7 μm) were washed with PBS and blocked in PBS containing 5% serum, 0.1% Triton X-100, and 1% BSA. Primary and secondary antibodies were diluted in blocking solution. For primary antibody staining, anti-EVA was from Proteintech (rabbit polyclonal, used at 1:100 dilution); rabbit anti-C5b-C9 complement complex and rabbit anti-myelin basic protein were from Abcam; rat anti-CD11b (M1/70), CD4 (GK1.5), CD5 (53-7.3), and rabbit isotype controls were from eBioscience. Alexa dye labeled secondary antibodies (donkey anti-rat and anti-rabbit) and anti-mouse IgG were from Invitrogen.

*Fluorescent microscopy.* Fluorescent images were acquired and analyzed using a Zeiss Axiovert 200 fluorescent microscope equipped with an Axiocam-MRm CCD camera; 10x, 20x and 40x objectives (Zeiss); and Axiovision version 4.8 software.

For quantitative analysis of complement staining, whole spinal cords from mice immunized with MOG^35-55^ were analyzed (onset of peak disease, day 19), and regions of inflammatory infiltrates, as defined by DAPI staining and analysis of serial sections stained for immune cell markers, were imaged using a 40x objective (Zeiss). Three complete sections from at least three separate spinal cords were analyzed for each condition. Images were analyzed using the AutoMeasurement component of Axiovision software, and the area of complement staining was expressed as densitometric counts in relative fluorescent units per μm^2^.

*Flow cytometry.* Cell preparation, staining, and analysis for flow cytometry were performed as described previously [3,4]. For mononuclear single cell preparations from spinal cord, a modification of the protocol of Babb *et al.*, 2000 was used [5]. Mice were deeply anesthetized with ketamine-xylazine and perfused with 50 ml of ice-cold PBS. Tissue was minced, dissociated with the plunger of a 5 ml syringe, and strained through a 0.1 mm filter. Cells were isolated following 2 washes (2118 x g for 15 min) in 37% Percoll in RPMI media.

The following antibodies were used for staining of single cell preparations: anti-EVA (Proteintech, rabbit polyclonal, used at 1:100 dilution); Alexa 488 goat anti-mouse IgG (Invitrogen); and APC-labeled anti- CD19, APC-eFluor 780 CD4 (GK1.5), PE CD8 (53-6.7), PE CD5 (53-7.3), eFluor 450 CD11b (M1/70) (eBioscience). For EVA staining, cells were fixed and permeabilized utilizing reagents from a commercially available kit (Becton-Dickinson), and intracellular staining was performed with anti-EVA antibody followed by Alexa 488 labeled donkey anti-mouse secondary antibody (Invitrogen). Data acquisition was performed on a LSRII flow cytometer (Becton-Dickinson) at the University of Wisconsin Carbone Cancer Center core facility at the Wisconsin Institute for Medical Research (WIMR).

*Data analysis*. Data were analyzed by using Axiovision 4.8 (Zeiss) and FlowJo (Treestar) software. Statistical analysis (SEM calculation and *t*-test) was performed using Kaleidagraph 4.1 (Synergy).

*ELISA.* Detection of serum autoantibody was performed using a SensoLyte anti-mouse MOG (1-125) IgG quantitative ELISA kit (Anaspec) per the manufacturer's protocol.

*RT-PCR and quantitative PCR***.** Reverse transcription weas performed with Superscript II (Gibco). Quantitative PCR was performed using TaqMan primers (FAM-labeled) commercially obtained (Applied Biosystems), and samples were run on a SmartCycler [2,4]. Samples were normalized to GAPDH Ct values for each experiment and expressed as normalized copy number relative to GAPDH.

References

1. Tang T, Li L, Tang J, Li Y, Lin WY, et al. (2010) A mouse knockout library for secreted and transmembrane proteins. Nat Biotechnol 28: 749-755.

2. Carrithers M, Dib-Hajj S, Carrithers L, Tokmoulina G, Pypaert M, et al. (2007) Expression of the voltage-gated sodium channel NaV1.5 in the macrophage late endosome regulates endosomal acidification. J Immunol 178: 7822-7832.

3. Carrithers MD, Carrithers LM, Czyzyk J, Henegariu O (2007) Characterization of a severe parenchymal phenotype of experimental autoimmune encephalomyelitis in (C57BL6xB10.PL)F1 mice. J Neuroimmunol 187: 31-43.

4. Wojcik E, Carrithers LM, Carrithers MD (2011) Characterization of epithelial V-like antigen in human choroid plexus epithelial cells: Potential role in CNS immune surveillance. Neurosci Lett.

5. Brabb T, von Dassow P, Ordonez N, Schnabel B, Duke B, et al. (2000) In situ tolerance within the central nervous system as a mechanism for preventing autoimmunity. J Exp Med 192: 871-880.
